# Programmable Guest‐Induced 5‐Phase‐Transition Topological Reconstructions of Cobalt Formate Metal–Organic Frameworks

**DOI:** 10.1002/advs.77629

**Published:** 2026-09-11

**Authors:** Zhiyu Tao, Jingjian Li, Jing Ling, Tianxiang Chen, Wing‐Yiu Yu, Cong Lin, Tsz Woon Benedict Lo

**Affiliations:** ^1^ Department of Chemistry The Hong Kong Polytechnic University Hung Hom Hong Kong People's Republic of China; ^2^ PolyU‐Daya Bay Technology and Innovation Research Institute The Hong Kong Polytechnic University Huizhou Guangdong People's Republic of China; ^3^ The Hong Kong Polytechnic University Shenzhen Research Institute The Hong Kong Polytechnic University Shenzhen People's Republic of China; ^4^ Department of Physics and Materials The Hong Kong Polytechnic University Hung Hom Hong Kong People's Republic of China

**Keywords:** amorphous solid, blueprint, chemoselectivity, diamondoid, formate, materials science, molecule, nanotechnology

## Abstract

Structural dynamics in metal–organic frameworks (MOFs) typically rely on elaborate, flexible organic linkers. Here, we challenge this paradigm by demonstrating a rare, reversible 5‐phase‐transition cycle in a minimalist cobalt formate MOF system. Central to this evolution is the conceptual reframing of the amorphous state (**Co‐Amor**); rather than representing a structural dead‐end, **Co‐Amor** functions as a programmable, high‐energy reactive hub that lowers reorganization barriers. We show that specific guest molecules selectively direct the reconstruction of this amorphous matrix into distinct crystalline architectures, including chiral (**Co‐Hex**), perovskite‐like (**Co‐Trig**), diamondoid (**Co‐Mono1**), and hydrated (**Co‐Mono2**) frameworks, effectively ‘reprogramming’ the material's underlying lattice physics. The generality of the guest‐molecule selectivity is verified by **Co‐Hex** and **Co‐Trig**, which have different crystalline structures but share the same phase‐transition behavior, thereby further highlighting the adaptability and importance of this system. Most notably, we uncover a rare gas‐solid reconstruction where CO_2_ acts as a morphogenic trigger, inducing a gradual amorphous‐to‐crystal transition to the rigid **Co‐Mono1** phase. This chemoselectivity originates from a specific C–H···O hydrogen‐bond complementarity between the formate channel walls and CO_2_. This work highlights the untapped potential of amorphous intermediates in directing structural reconfigurations, offering a blueprint for designing highly adaptive materials from the simplest molecular building blocks.

## Graphical abstract

A reversible five‐phase topological cycle is achieved in a minimalist cobalt formate metal–organic framework. The amorphous phase functions as a programmable reactive hub, selectively reconstructing into four distinct crystalline architectures through cationic templates, humidity, or CO_2_. Remarkably, CO_2_ triggers a rare amorphous‐to‐crystal transition via specific formate–CO_2_ hydrogen‐bond recognition, enabling guest‐programmed pore engineering.

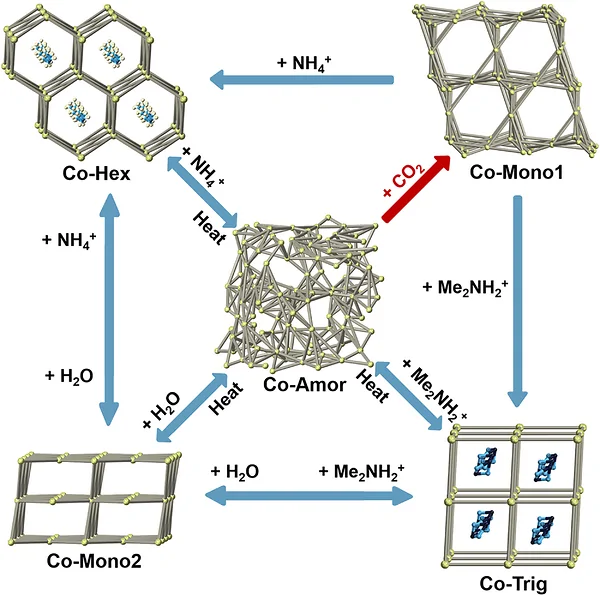

## 1 Introduction

The design of stimuli‐responsive ‘smart’ materials has transitioned from simple binary switches to the pursuit of programmable structural plasticity [1, 2, 3]. Within the field of metal–organic frameworks (MOFs), this evolution is characterized by the movement beyond discrete ‘gate‐opening’ or ‘breathing’ transitions toward systems capable of navigating a dense landscape of multiple metastable states [4, 5]. While significant progress has been made using ‘top‐down’ approaches (e.g., incorporating elaborate, sterically demanding, or photo‐active linkers to drive structural change), a fundamental question remains: can high‐order structural complexity be genuinely ‘programmed’ through external stimuli?

Structural reconstruction, involving the coordinated breaking and reforming of metal–ligand bonds, represents a profound form of MOF dynamics [6, 7]. Unlike mechanical compliance (e.g., the MIL‐53 family) or local gate‐opening (e.g., ZIF‐8) [8, 9, 10, 11, 12, 13, 14], reconstructive transitions allow for complete topological reconfiguration, potentially unlocking vastly different physicochemical properties within a single material system [15]. However, these transitions are traditionally difficult to control: they often follow predetermined, single‐pathway trajectories or result in irreversible collapse into ‘dead’ amorphous phases [16, 17, 18, 19]. The ability to harness an amorphous state as a functional reactive hub, rather than a decomposition ‘end’, and to navigate between multiple crystalline topologies would represent a genuine shift in the synthesis of adaptive materials. In this context, the role of guest molecules would evolve from a passive pore occupant to an active morphogenic agent that directs the framework's reconstruction pathway.

The formate ligand (HCOO^−^) offers a unique, yet underutilized, platform for exploring this hypothesis. As the simplest carboxylate, its extreme coordination versatility (*syn‐syn*, *anti‐anti*, and *syn‐anti* bridging modes) and low rotational barriers provide the necessary degrees of freedom for complex structural rearrangements under mild energetic perturbations [20, 21]. While formate‐based frameworks have been studied for their ferroelectric and magnetic properties [22, 23, 24, 25], and more recently for gas adsorption and separation exploiting their intrinsic microporosity [26, 27, 28], their high‐order structural plasticity, particularly multi‐pathway phase interconversions, remains relatively unexplored, often overlooked due to the perceived simplicity of the formate linker.

**FIGURE 1:**
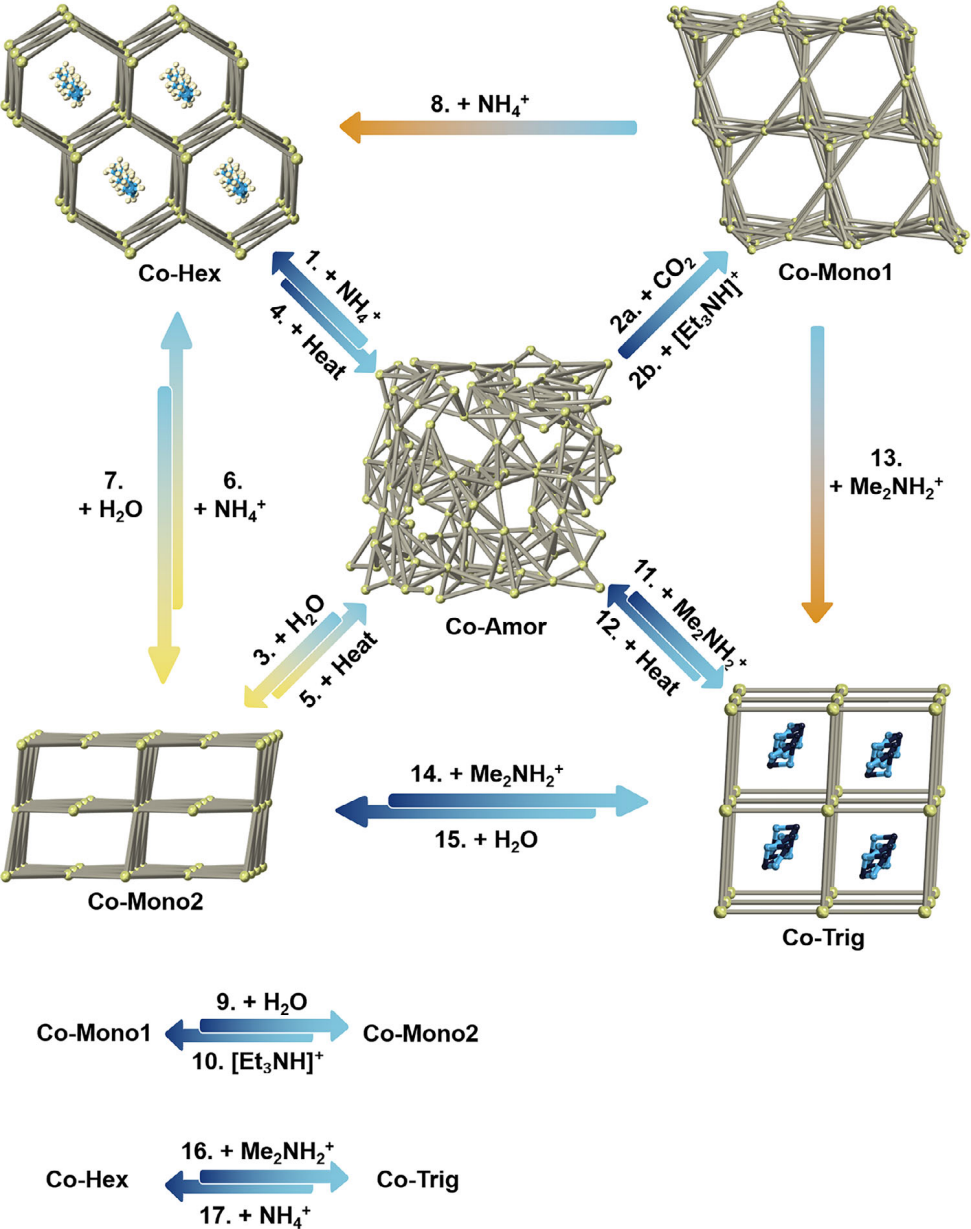
Reversible 5‐phase‐transition cycle among five Co‐formate MOFs. Pathways 1, 6, 8 and 17: NH_4_^+^ immersion; pathway 2a: CO_2_; pathways 2b and 10: Et_3_NH^+^ immersion; pathways 3, 7, 9 and 15: humid air; pathways 4, 5 and 12: heating; pathways 11, 13, 14 and 16: Me_2_NH_2_^+^ immersion.

Herein, we report the discovery of a rare reversible 5‐phase‐transition cycle in a cobalt formate system. By interconnecting five distinct structural states, namely, [NH_4_][Co(HCOO)_3_] (a chiral hexagonal framework, ‘**Co‐Hex**’), [(CH_3_)_2_NH_2_][Co(HCOO)_3_] (a trigonal perovskite‐like framework, ‘**Co‐Trig**’), Co(HCOO)_2_ (a monoclinic diamondoid framework, ‘**Co‐Mono1**’), Co(HCOO)_2_·2H_2_O (a monoclinic dihydrate, ‘**Co‐Mono2**’), and Co(HCOO)_2_ (an amorphous phase, ‘**Co‐Amor**’), we demonstrate that this minimalist cobalt formate MOF framework can be programmed to navigate a complex phase‐space via temperature, humidity, and chemical stimuli (Figure 1). Central to this work is the concept of guest‐induced reconstruction, identifying **Co‐Amor** as an adaptive ‘switchboard’ where the absence of long‐range order enables maximum sensitivity to chemical cues. We demonstrate a rare CO_2_‐induced amorphous‐to‐crystal transition yielding **Co‐Mono1** and, through density functional theory, in situ Raman spectroscopy, and systematic probe‐gas screening, establish that a specific C–H···O hydrogen‐bond recognition, rather than quadrupole moment or kinetic diameter, governs this selectivity. This contrasts sharply with the framework's response to other gas molecules, such as N_2_, H_2_, C_2_H_2_, and C_3_H_8_, revealing a high degree of chemoselective recognition. The cation‐templating strategy of **Co‐Hex** and **Co‐Trig** broadens the generality and compatibility of **Co‐Amor** as a reactive hub. By utilizing time‐resolved synchrotron X‐ray powder diffraction (PXRD) and synchrotron X‐ray pair distribution function (XPDF) analysis, we map the guest‐induced structural modulations within this reversible 5‐phase‐transition system. This work establishes a new blueprint for materials design, where we can effectively leverage the inherent conformational flexibility of simple connectors and the kinetic accessibility of amorphous intermediates to achieve sophisticated, multi‐pathway responsiveness that rivals the adaptive behavior of complex biological systems.

## 2 Results

### 2.1 Synthesis and Structural Characterization

To explore the structural plasticity of the cobalt formate system, we employed high‐yield synthetic routes to the distinct coordination chemistry. Specifically, **Co‐Hex** was obtained as violet‐red bipyramidal crystals via a modified diffusion method [22]; the neutral **Co‐Mono1** was prepared as pink plates via a solvothermal method [29, 30]; the pale pink hydrated **Co‐Mono2** was obtained through exposure of any anhydrous phase to humid air. In contrast, the purple **Co‐Amor** was generated via the thermal de‐templating of **Co‐Hex** or dehydration of **Co‐Mono2** at 200°C under vacuum (see Materials and Methods for the detailed synthesis procedures). **Co‐Trig**, an additional cation‐templated phase examined here for comparison, was synthesized as red‐pink cuboidal particles via a modified solvothermal method [20, 31]. Elemental analysis confirmed the high purity of each phase (Table S1). Notably, the nitrogen content in **Co‐Mono2** was negligible (<0.44%), showing the successful removal of the ammonium template and the formation of pure cobalt formate variants. Their chemical compositions and thermal stabilities were studied by thermogravimetric analysis (Figure S1). Their different initial particle morphologies, as observed by scanning electron microscopy, are shown in Figure S2, and their macroscopic optical appearances are presented in Figure S3.

**FIGURE 2:**
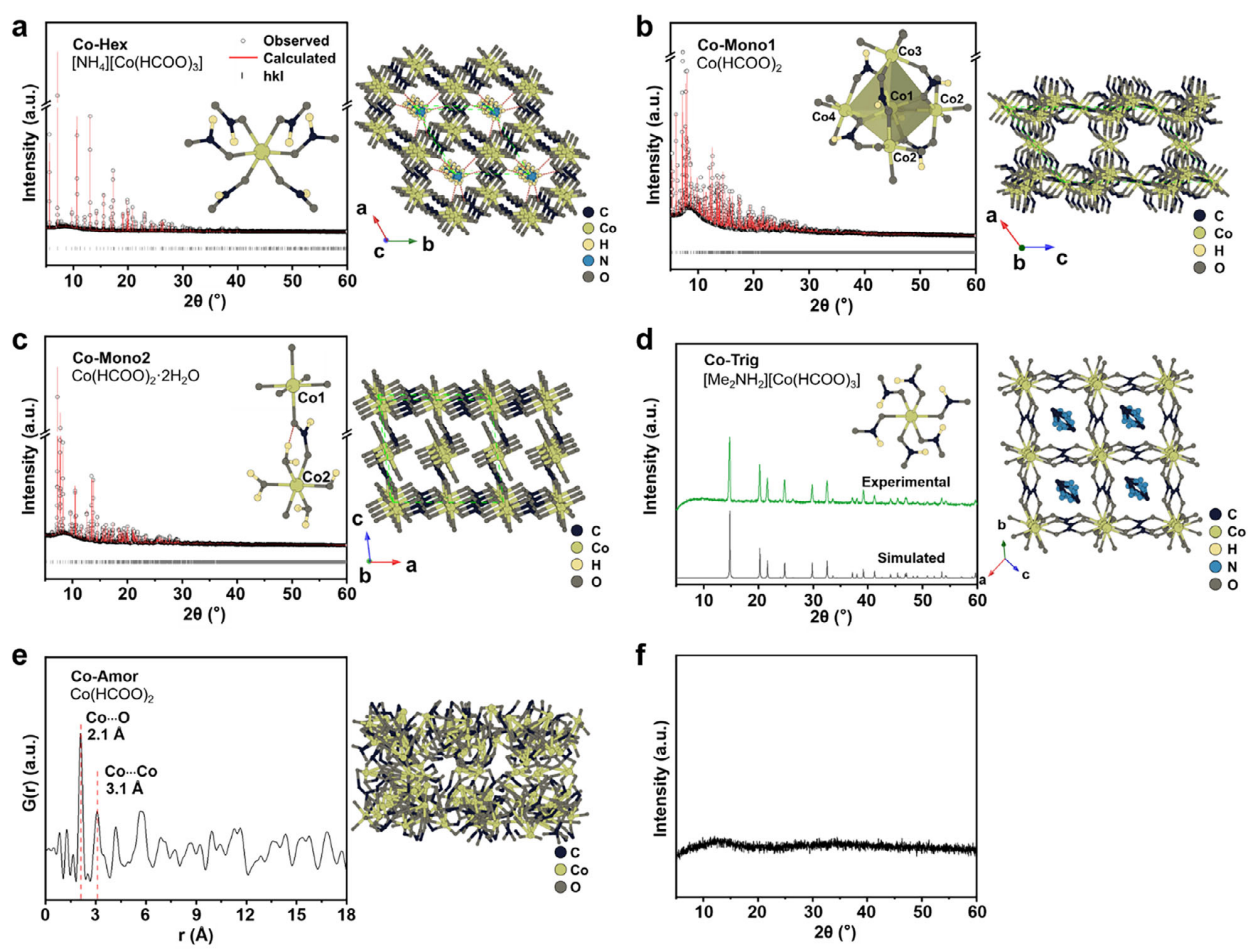
Phase identification and structure of the Co‐formate landscapes. Pawley refinement profiles of synchrotron PXRD data (λ = 0.61992 Å; E = 20 keV) and framework topologies for (a) **Co‐Hex**, (b) **Co‐Mono1**, and (c) **Co‐Mono2**, with corresponding coordination environments shown in the insets and refinement details and crystallographic parameters provided in Table S2. (d) Laboratory PXRD pattern of **Co‐Trig** (Cu anode; compared with the profile simulated from the reported single‐crystal structure) together with its framework topology. At room temperature, these cations are dynamically disordered over three symmetry‐equivalent orientations, and their hydrogen atoms are therefore omitted from the structural model for clarity. (e) XPDF of **Co‐Amor**, with a schematic illustration of its amorphous structure. (f) Featureless laboratory PXRD pattern of **Co‐Amor**, confirming the absence of long‐range order.

Chemical consistency of the formate backbone across the Co‐formate structural landscape was validated by Fourier‐transform infrared (FTIR) spectroscopy (Figure S4). Characteristic vibrational fingerprints were observed across all phases, including the asymmetric carboxylate stretch (ν(OCO)_as_) at 1591 cm^−1^, the symmetric carboxylate stretch (ν(OCO)_s_) at 1373 cm^−1^, and the Co–O lattice vibrations at 570 and 807 cm^−1^ [24, 29]. Complementing these spectroscopic data, high‐resolution synchrotron powder X‐ray diffraction (PXRD) coupled with Pawley refinement revealed the phase purity and long‐range structural integrity of **Co‐Hex**, **Co‐Mono1**, and **Co‐Mono2**, whose crystal structures are briefly described below (Figure 2). As **Co‐Trig** serves only as a complementary cation‐templated phase, its phase purity was verified at the laboratory level by matching its experimental PXRD pattern against the profile simulated from the reported single‐crystal structure (Figure 2d).

- **Co‐Hex** adopts a chiral hexagonal structure with a space group of *P*6_3_22 (*a* = 7.306 Å, *c* = 8.186 Å, *V* = 378.5 Å^3^), featuring an anionic (4^9^6^6^) framework. Each Co^2+^ center is octahedrally coordinated by six *anti‐anti* formate bridges, creating helical one‐dimensional channels (∼6.8 Å) along the *c*‐axis that host disordered NH_4_^+^ cations stabilized by N–H···O hydrogen bonds (Figure 2a, inset) [23].
- **Co‐Mono1** involves a total topological reconfiguration into a monoclinic diamondoid network with a space group of *P*2_1_/*c* (*a* = 11.324 Å, *b* = 9.950 Å, *c* = 18.347 Å, *β* = 127.193°, *V* = 1656 Å^3^). The framework is stabilized by a combination of *syn‐syn/anti* modes, resulting in zigzag channels (∼5–6 Å) and a more condensed unit cell (Figure 2b, inset) [25, 29].
- **Co‐Mono2** represents a dense, non‐porous state with a monoclinic lattice and a space group of *P*2_1_/*c* (*a* = 8.682 Å, *b* = 7.171 Å, *c* = 9.278 Å, *β* = 97.247°, *V* = 572.8 Å^3^). There are two Co coordination spheres in this structure, where the Co1 site maintains a three‐dimensional connectivity via the *anti‐anti* bridges like **Co‐Hex**. In contrast, the Co2 site is coordinated with four terminal water and two formate ligands via the *syn‐anti* bridges (Figure 2c, inset) [32].
- **Co‐Trig** crystallizes as a perovskite‐like trigonal framework (space group *R*3̅c; *a* = 8.199 Å, *c* = 22.224 Å, *V* = 1294 Å^3^) [20, 31]. Each Co^2+^ center adopts a trigonally distorted octahedral (CoO_6_) geometry and is bridged to six neighbors by *anti‐anti* formate bridges, generating an anionic NaCl‐type [Co(HCOO)_3_]^−^ framework whose pseudo‐cubic cavities host the dimethylammonium (Me_2_NH_2_^+^) cations. In contrast to the four N–H donors of NH_4_^+^ in **Co‐Hex**, the Me_2_NH_2_^+^ cation provides only two N–H donors, which anchor it to the framework formate oxygens through N–H···O hydrogen bonds (Figure 2d).
- (f) While **Co‐Amor** lacks long‐range periodicity, as seen in its laboratory PXRD pattern (Figure 2f), our synchrotron X‐ray pair distribution function (XPDF) analysis (λ = 0.16520 Å, E = 75.051 keV) shows the local ordering of Co···formate interactions (Figure 2e). The observed G(r) data show apparent peaks at approximately 2.1 and 3.1 Å, corresponding to the first‐shell Co–O and Co···Co distances, respectively (Figure 2e). These peaks match the local coordination motifs of the other crystalline phases (as shown in the simulated XPDF profiles of other MOFs in the Figures S5–S7), indicating that the primary Co(HCOO)_2_ building units remain intact despite the loss of long‐range order beyond 10 Å. This retention of short‐range structural integrity constitutes a building‐block memory: the primary Co(HCOO)_2_ coordination units are preserved even though all long‐range connectivity is erased. Crucially, **Co‐Amor** does not store any single preferred lattice; rather than a ‘topological memory’, it behaves as a reservoir of intact building blocks whose ultimate topology is programmed by the incoming guest, enabling it to serve as a programmable precursor to multiple crystalline endpoints.

### 2.2 Guest‐Induced Multi‐Pathway Phase Transitions

To probe the guest‐induced controllability of the Co‐formate system, we used the amorphous **Co‐Amor** phase as the adaptive switchboard. We monitored its structural evolution by time‐resolved PXRD upon exposure to specific guest species (Figure 3). Experimental details and the PXRD dataset for the complete reversible 5‐phase‐transition cycle are provided in Figures S8–S19.

**FIGURE 3:**
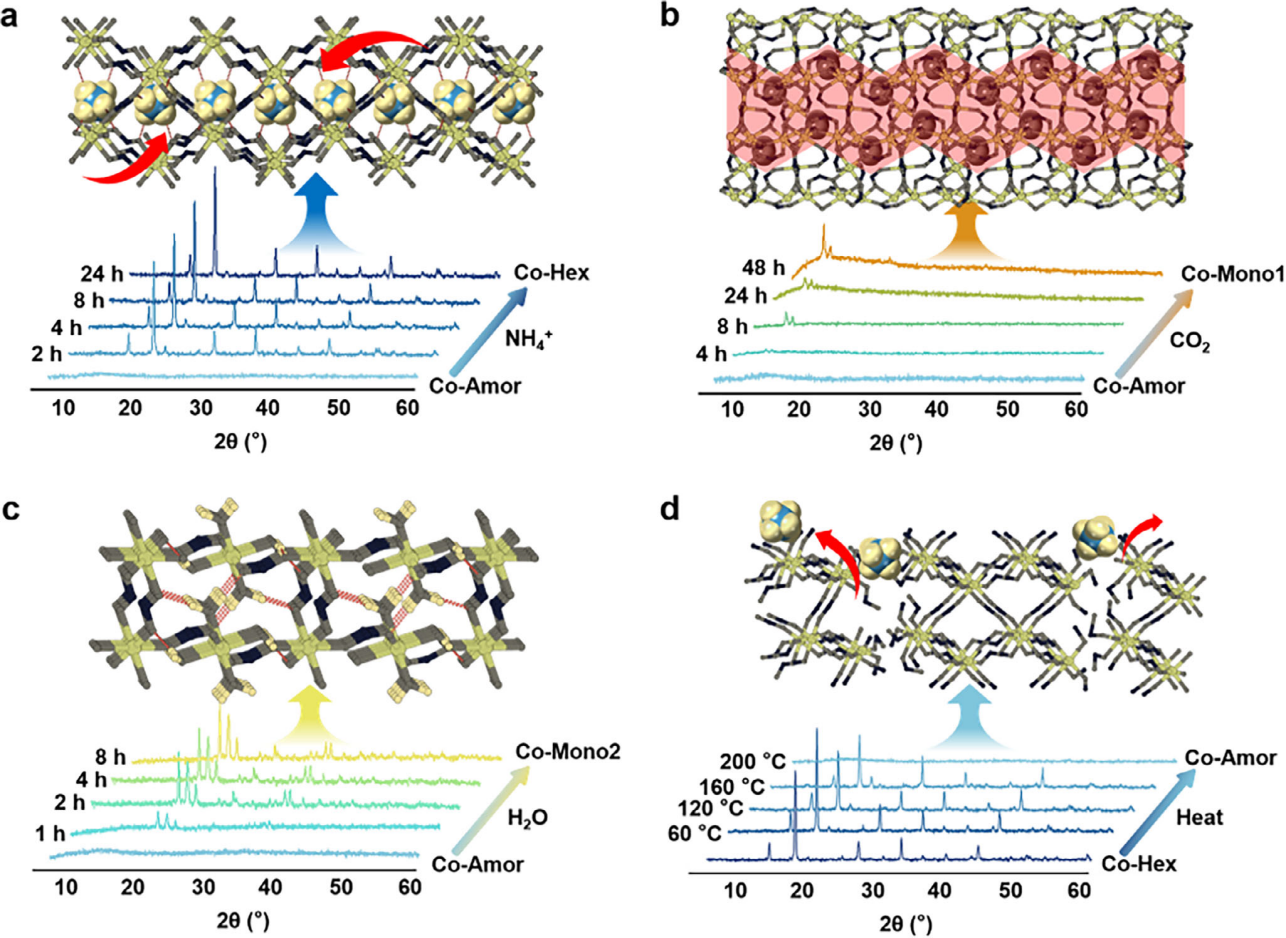
Structural reconstruction pathways of the **Co‐Amor** tracked by time‐resolved PXRD measurements. (a) Guest‐induced crystallization of the chiral **Co‐Hex** phase upon immersion in NH_4_HCOO/methanol. (b) Permanent gas‐solid reconstruction to the diamondoid **Co‐Mono1** induced by CO_2_, with the red zone representing the one‐dimensional channel along the *b*‐axis. (c) Coordination‐driven hydration to the **Co‐Mono2** phase under humid air. (d) Thermal de‐templating and amorphization of **Co‐Hex**.

We first investigated the guest‐induced reconstruction directed by ammonium ions (NH_4_^+^, which was also used in the synthesis of **Co‐Hex**). The chiral **Co‐Hex** phase can be reconstructed from **Co‐Amor** (Pathway 1, Figure 3a), which illustrates a highly ordered cation‐templated assembly. Upon immersion in NH_4_HCOO/methanol, the featureless PXRD of **Co‐Amor** shows clear changes within the first few hours. Gradual emergence and sharpening of the characteristic Bragg peaks of **Co‐Hex** were observed, reaching high crystalline maturity within 24 h. Mechanistically, this phase transition can be attributed to a hydrogen‐bond‐mediated crystallization. Specifically, the NH_4_^+^ cation acts as a tetrahedral structural anchor, utilizing its four N–H···O hydrogen bonds to direct the disordered formate bridges into the specific *anti‐anti* bridging mode required for the hexagonal (4^9^6^6^) topology. This shows that the recovered topology is dictated by the guest rather than by an intrinsic memory of the parent lattice: the NH_4_^+^ cation supplies the exact geometric information needed to reorganize the preserved local Co‐formate building units into the hexagonal (4^9^6^6^) net. Because the same amorphous precursor can instead be directed toward trigonal, diamondoid, or hydrated frameworks by other guests (vide infra), the process is best described as a building‐block memory combined with guest‐programmed topology, rather than a return to a single remembered state.

To verify the generality of this cation‐templating mechanism, we turned to another reported cation‐filled cobalt formate framework [20]. We replaced NH_4_^+^ with dimethylammonium cations (Me_2_NH_2_^+^). Immersion of **Co‐Amor** in Me_2_NH_2_HCOO/methanol (Pathway 11, Figure S19a) yielded the trigonal perovskite‐like (*R*3̅c) **Co‐Trig** phase within 24 h, as confirmed by the emergence of its characteristic Bragg reflections. As mentioned, Me_2_NH_2_^+^ also plays a structural anchor through its N–H···O hydrogen bonds, encoding specific geometric information into the recrystallized framework. Additionally, **Co‐Trig** and **Co‐Hex** are fully interconvertible: immersion of **Co‐Trig** in NH_4_HCOO/methanol produces **Co‐Hex** (Pathway 17, Figure S19d), while immersion of **Co‐Hex** in Me_2_NH_2_HCOO/methanol yields **Co‐Trig**, establishing a direct cation‐exchange pathway between the two templated phases. The complete equivalence of **Co‐Trig** and **Co‐Hex** in their phase‐transition behavior confirms that the identity of the protonated amine cation selectively encodes the resulting framework topology. At the same time, the underlying amorphous‐to‐crystalline reconstruction mechanism remains invariant.

Another specificity of these hydrogen‐bonding interactions is highlighted by substituting NH_4_^+^ with the bulkier Et_3_NH^+^ (Pathway 2b and 10, Figures S10 and S18). The ethyl groups impose steric bulk that prevents hexagonal channel formation, instead directing the reconstruction toward the diamondoid **Co‐Mono1** with wider zigzag channels. This demonstrates that the cation geometry directly encodes the resulting topology. In contrast, the immersion of Et_3_N/MeOH yields no structural change, confirming that a protonated cationic template with hydrogen bonding is the prerequisite for reconstruction [21, 23]. Extending this survey to other alkylammonium cations did not expand the phase landscape: diethylammonium (Et_2_NH_2_^+^) likewise reconstructs **Co‐Amor** into the diamondoid **Co‐Mono1** (as for Et_3_NH^+^), whereas the bulkier tert‐butylammonium (^t^BuNH_3_^+^) induced no crystallization under identical conditions, delineating a practical size window for cation‐directed reconstruction.

One critical piece of evidence for the programmable control is the CO_2_‐induced reconstruction of **Co‐Amor** into **Co‐Mono1** (Pathway 2a, Figure 3b). To the best of our knowledge, this represents a rare (if not the first) example of a CO_2_‐induced amorphous‐to‐crystalline transition observed in MOFs. While the framework remains inert under N_2_, exposure to 1 atm of CO_2_ at room temperature initiates a gradual crystallization of the monoclinic diamondoid **Co‐Mono1** state over 24–48 h (Figure 3b), the slow kinetics reflecting the high energetic cost of reconfiguring the entire three‐dimensional coordination network into the *syn‐syn/anti* diamondoid lattice. To identify the molecular driving force, we performed density functional theory (DFT) calculations on the **Co‐Mono1** framework with adsorbed CO_2_ (Figure S20c). The optimized geometry reveals a specific C–H(formate)···O═C═O hydrogen bond (d(H···O) = 2.751 and 2.855 Å) with a binding energy of 0.79 eV per CO_2_. At the same time, the adsorbed molecule retains a near‐linear geometry (O═C═O angle = 178.773°), confirming a physisorptive, non‐activating interaction. CO_2_ thus acts as a hydrogen‐bond acceptor toward the weakly acidic formate C–H donors lining the channel walls. This specific molecular‐recognition event supplies the enthalpic driving force for the amorphous‐to‐crystalline reorganization [33].

We further utilized in situ Raman spectroscopy to investigate this phenomenon (Figure S20). During exposure of **Co‐Mono1** to CO_2_ (0–60 min), a band at ∼1280 cm^−1^, the lower‐frequency component of the Fermi dyad of adsorbed CO_2_, appears within minutes and thereafter remains unchanged, while the framework formate ν(OCO) mode red‐shifts slightly from 1394 to 1389 cm^−1^. This subtle red‐shift is the vibrational fingerprint of the C–H···O═C═O interaction, and the time‐invariant CO_2_ band confirms fast, reversible physisorption, consistent with the DFT results (Figure S20c). Complementary Raman measurements on **Co‐Amor** under CO_2_ (0–36 h, same sample spot) capture the crystallization itself: the broad, featureless amorphous profile progressively develops sharp peaks matching the crystalline **Co‐Mono1** lattice modes, providing direct spectroscopic evidence of the CO_2_‐triggered structural ordering [34].

The chemoselectivity of this reconstruction was probed by exposing **Co‐Amor** to N_2_, H_2_, C_2_H_2_, and C_3_H_8_ under identical conditions (1 atm, 25°C, 48 h; Figure S21 and Table S3); none induced any detectable crystallization. Despite C_2_H_2_ possessing a larger quadrupole moment than CO_2_ (Q(C_2_H_2_) = 7.5 × 10^−40^ C·m^2^ vs. Q(CO_2_) = 4.3 × 10^−40^ C·m^2^) and an identical kinetic diameter (3.3 Å), it fails to trigger the phase transition. This rules out, to a large extent, quadrupolar strength and molecular size as the operative factors and points instead to hydrogen‐bond directionality: CO_2_ is an exclusive H‐bond acceptor that complements the formate C–H donors of the channel walls, whereas C_2_H_2_ is an H‐bond donor (via its acidic ≡C–H) that creates a donor–donor mismatch. Only a guest satisfying this complementary host–guest geometry can lower the reorganization barrier enough to convert the disordered **Co‐Amor** matrix into the ordered **Co‐Mono1** sieve. Unlike the reversible breathing of flexible MOFs, this is a permanent, guest‐encoded transition [35].

We subsequently studied the sensitivity of **Co‐Amor** to humidity (Pathway 3, Figure 3c), showing that the characteristic reflections of **Co‐Mono2** emerge gradually over 8 h upon exposure to humid air (relative humidity of ∼60%). A coordinative competition characterizes this transition. Here, water molecules act as the competitive ligands, coordinating directly to the Co^2+^ centers (four water ligands on the Co2 site). In contrast, immersion in non‐coordinating solvents (such as methanol, ethanol, acetone, and N,N‐dimethylformamide) leaves the amorphous state unchanged. This indicates that the transition is facilitated by an extensive inter‐framework hydrogen‐bonding network between coordinated water and formate oxygens, acting as a ‘structural stabilizer’ that tilts the CoO_6_ octahedra [32]. The complete loss of N_2_ accessibility (see the adsorption discussion below) correlates perfectly with the final PXRD pattern of the dense **Co‐Mono2** phase, where water molecules effectively block the channels.

Conversely, we can reset all the crystalline phases back to their amorphous **Co‐Amor** hub by simply removing the guest inductions via thermal stimuli. For instance, we monitored the **Co‐Hex** to **Co‐Amor** transition using time‐resolved PXRD under heating (Figure 3d, Pathway 4) and temperature‐programmed desorption coupled with mass spectrometry (TPD‐MS, Figure S22). Specifically, heating **Co‐Hex** to 180°C causes a systematic broadening and eventual disappearance of the Bragg reflections, directly tracking the thermal de‐templating. Thermogravimetric analysis also supports this, with 28.3% mass loss at 175°C (equivalent to one NH_4_HCOO molecule per formula unit; Figure S1a), yielding **Co‐Amor**. TPD‐MS further confirms the simultaneous release of NH_3_ (m/z of 17) and HCOOH (m/z of 46). This coordinated desorption signals the thermal de‐templating of **Co‐Hex**, where its N–H···O interactions are cleaved, and the ordered *anti‐anti* bridging network is disrupted.

Similarly, the dehydration of **Co‐Mono2** (Pathway 5, Figure S13) was identified as involving two H_2_O loss events at 75°C and 160°C (Figure S1c), leading to framework collapse to **Co‐Amor** at ∼200°C. In contrast, **Co‐Mono1** exhibits significantly higher coordinative robustness, resisting decomposition until ∼300°C. This disparity in stability confirms that **Co‐Hex**, **Co‐Trig,** and **Co‐Mono2** are guest‐stabilized kinetic phases, whereas **Co‐Mono1** represents the anhydrous thermodynamic sink.

The reversible transitions between these crystalline and amorphous phases match the paradigm of recoverable collapse [36, 37]. In this regime, the phase loses long‐range periodicity without compromising the chemical integrity of its primary building units, which aligns with our XPDF result (Figure 2e). A fundamental entropy‐enthalpy competition governs this structural reversibility: the low‐entropy crystalline phase is accessible only when stabilized by the enthalpic gain from guest‐framework interactions. Specifically, the system favors a disordered phase (entropy‐favored **Co‐Amor**) unless a guest molecule or a template, such as NH_4_^+^, H_2_O, or CO_2_, provides sufficient enthalpy via hydrogen bonding or quadrupolar interactions to overcome the entropic penalty of long‐range organization. In this picture, the amorphous phase encodes the building units while each guest encodes the target topology, so that ‘memory’ and ‘programmability’ are complementary rather than contradictory. In the absence of these molecular instructors, the phase remains in the high‐entropy amorphous reservoir, chemically available for subsequent guest‐induced reconstruction [38].

The versatility of this cobalt formate system is encapsulated in a reversible 5‐phase‐transition cycle that interconnects all five phases (Figure 1). Beyond the primary transformations, the cycle closes bidirectionally: **Co‐Mono2** and **Co‐Mono1** can be reverted to the chiral **Co‐Hex** via NH_4_^+^ templating (Pathways 6 and 8), humid air drives the anhydrous phases transitioning toward the hydrated **Co‐Mono2** (Pathways 3, 7, and 9), and Et_3_NH^+^ directs the dense **Co‐Mono2** transformation toward the porous **Co‐Mono1** (Pathway 10). **Co‐Trig** also undergoes phase transition pathways among the other four cobalt formate structures (Figure S19). Scanning electron micrograph tracking of two representative closed loops corroborates the reconstructive, rather than topotactic, nature of these transitions (Figure S23). In the cation‐templated loop, sharp‐edged **Co‐Hex** bipyramids progressively lose their facet definition and shrink upon amorphization to **Co‐Amor**; NH_4_^+^‐driven re‐crystallization restores sharp‐edged, well‐faceted crystals of the identical hexagonal phase (confirmed by PXRD) but with a distinct habit (pentagonal‐frustum termini bridging a pentagonal‐prism core) and a reduced size, indicating that the framework is rebuilt through the amorphous hub rather than retained as a single‐crystal skeleton. In the **Co‐Amor**‐centered loop, the 100–200 nm spheres of **Co‐Amor** transform under CO_2_ into ∼20 µm hexagonal plates indistinguishable from directly synthesized **Co‐Mono1**; subsequent hydration to **Co‐Mono2** fragments the plate surfaces into fine particulates, and thermal treatment restores the original 100–200 nm spherical morphology, demonstrating full morphological reversibility around the amorphous hub. These transitions, confirmed by PXRD gradients (Figure 3 and Figures S8–S19), demonstrate a level of dynamic reconfigurability previously unseen in the simple carboxylate systems.

The dramatic topological reconfigurations observed in this five‐phase cycle suggest that the cobalt formate framework serves as a platform for the programmable control of physical properties beyond gas adsorption. By transitioning between the **Co‐Hex**, **Co‐Trig**, **Co‐Mono1**, **Co‐Mono2**, and **Co‐Amor** states, the material effectively reprograms its underlying lattice physics. For instance, the magnetic ground state of cobalt systems is sensitive to Co–O–C–O–Co exchange pathways; thus, reconstructing the helical channels of **Co‐Hex** into the diamondoid network of **Co‐Mono1** significantly alters the magnetic exchange symmetry. More specifically, the **Co‐Hex** phase (*P*6_3_22) is a spin‐canted (weak) antiferromagnet below ∼9.8 K, arising from the Dzyaloshinskii–Moriya interaction across the non‐centrosymmetric *anti‐anti* formate bridges [22, 23]. In contrast, its cation‐templated congener **Co‐Trig** (*R*3̅c) is an established multiferroic, exhibiting canted weak ferromagnetism at ∼14.9 K together with an order–disorder paraelectric‐antiferroelectric transition near 165 K [20, 31]. Meanwhile, the centrosymmetric **Co‐Mono1** (*P*2_1_/c) is non‐ferroelectric and orders antiferromagnetically only below 2 K. This provides a rare mechanism for guest‐induced magnetic switching independent of traditional spin‐crossover centers [30]. Similarly, the dielectric response is tunable through phase‐specific guest interactions [39]. The presence of ordered water in **Co‐Mono2** versus mobile NH_4_^+^ cations in **Co‐Hex**, combined with the symmetry shift from the non‐centrosymmetric *P*6_3_22 to the centrosymmetric *P*2_1_/c, suggests switchable dielectric constants and potential guest‐triggered ferroelectric behavior. Ultimately, these transitions demonstrate how minimalist MOFs can translate molecular‐scale recognition into macroscopically useful physical responses.

### 2.3 Porosity and Adsorption Dynamics

**FIGURE 4:**
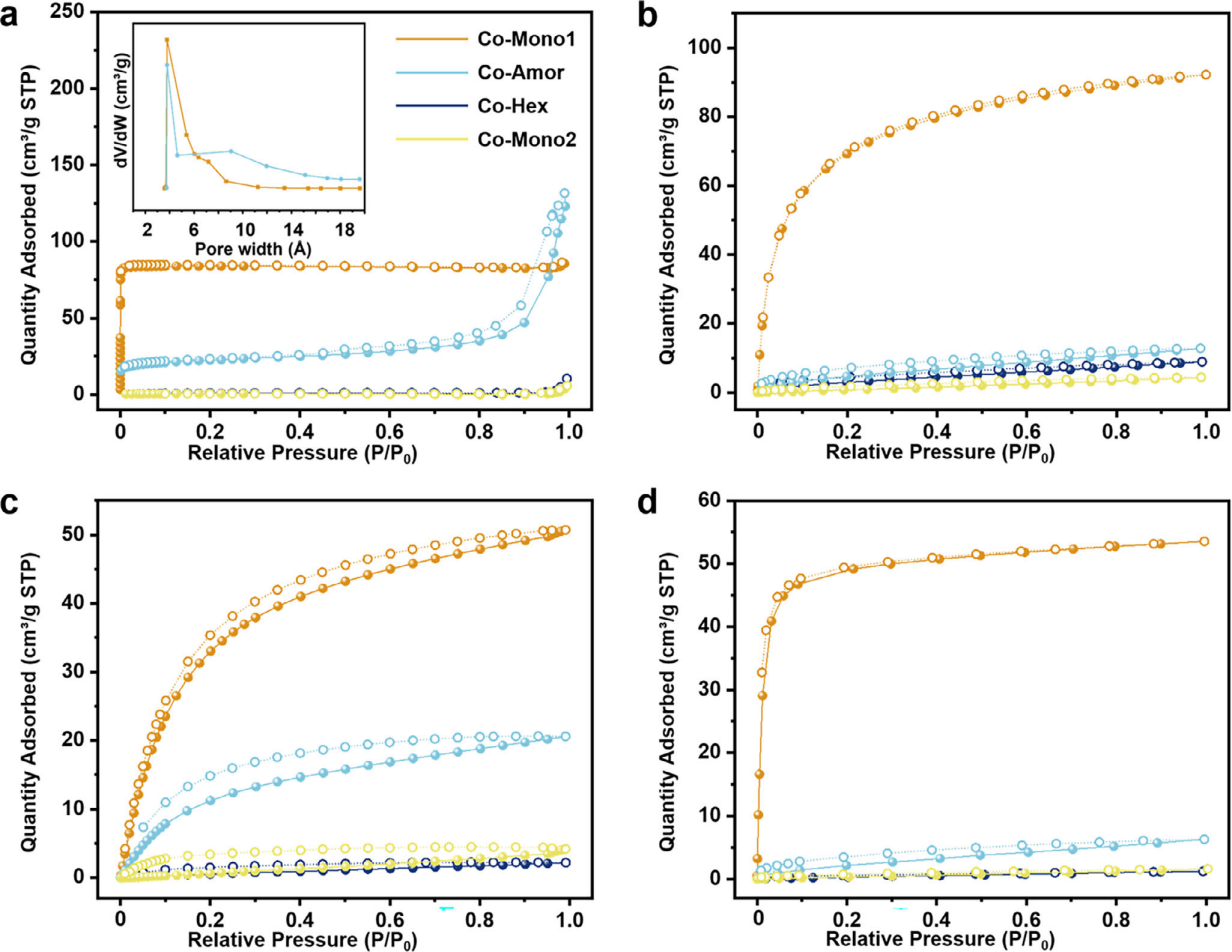
Gas adsorption isotherms. Adsorption (filled circles) and desorption (open circles) profiles for (a) N_2_ at 77 K, (b) H_2_ at 77 K, (c) CO_2_ at 273 K, and (d) C_2_H_2_ at 273 K, with micropore size distributions as the inset of (a). Data series are color‐coded as follows: orange for **Co‐Mono1**, cyan for **Co‐Amor**, dark blue for **Co‐Hex**, and yellow for **Co‐Mono2**.

The 5‐phase‐transition cycle of the cobalt formate landscape fundamentally redefines the pore architecture. To evaluate the functional consequences of these transitions, we first investigated the adsorption dynamics for N_2_, H_2_, and CO_2_ up to 1 bar. Specifically, the N_2_ isotherms at 77 K (Figure 4a and Figure S24) clearly distinguish the rigid microporosity of the three crystalline phases from the responsive **Co‐Amor**. **Co‐Mono1** displays the characteristic type I behavior with a maximum uptake of 86.3 cm^3^/g (at standard temperature and pressure (STP)) and a most probable pore size of ∼5.2 Å. This adsorption profile is consistent with its established crystal structure that possesses permanent, zigzag diamondoid channels (∼5.2 Å) [25, 29], serving as the rigid, high‐capacity limit of the anhydrous system. Conversely, **Co‐Hex**, **Co‐Trig,** and **Co‐Mono2** show negligible N_2_ uptake (with respective 10.5, 3.0, and 5.9 cm^3^/g uptake) due to the pore‐blocking cations in the former and hydration‐induced densification in the latter [23, 31, 32]. In stark contrast, **Co‐Amor** exhibits an apparent gate‐opening hysteresis, reaching a significantly higher uptake of 131.6 cm^3^/g and a most probable pore size of ∼8.4 Å. This reflects the inherent structural plasticity of the disordered phase, in which the amorphous matrix undergoes pressure‐induced expansion to accommodate guests, followed by kinetic trapping upon desorption, again evidencing the **Co‐Amor** role as the reactive hub. **Co‐Trig** exhibits negligible N_2_ uptake (3.0 cm^3^/g uptake) owing to the same cation‐blocking state as **Co‐Hex** (10.5 cm^3^/g uptake). This shows that **Co‐Trig** and **Co‐Hex** are functionally equivalent in their adsorption behavior, which was confirmed by CO_2_ uptake (2.7 and 2.2 cm^3^/g uptake). Given this equivalence, subsequent adsorption analysis focuses on four structurally distinct functional states, namely, **Co‐Hex**, **Co‐Mono1**, **Co‐Mono2**, and **Co‐Amor**.

To further explore the gating property of **Co‐Amor**, we utilized H_2_ as a smaller molecular probe (Figure 4b). While rigid **Co‐Mono1** excels with an uptake of 92.2 cm^3^/g, the uptake of **Co‐Amor** is limited to merely 12.8 cm^3^/g. This disparity suggests a size‐ or interaction‐dependent gating threshold, in which the small H_2_ molecule (kinetic diameter of 2.89 Å) and its weak van der Waals interactions are insufficient to overcome the expansion barrier of **Co‐Amor** [40]. These results show that **Co‐Amor** exhibits selective responsiveness, enabling precise molecular discrimination.

The CO_2_ isotherms at 273 K (Figure 4c) provide compelling evidence for the gas‐solid reconstruction (Pathway 2a). Typically, **Co‐Mono1** demonstrates a high affinity for CO_2_, with an uptake of 50.7 cm^3^/g, likely due to quadrupolar interactions with the formate‐lined pores [41]. In contrast, **Co‐Hex** and **Co‐Mono2** show poor CO_2_ adsorption, with uptakes of 2.2 and 4.5 cm^3^/g, respectively. Interestingly, the adsorption profile of **Co‐Amor** is highly anomalous. Rather than exhibiting the typical low capacity of a non‐porous amorphous solid, its CO_2_ uptake progressively increases, reaching a moderate 20.6 cm^3^/g. Notably, the adsorption isotherm is continuous, lacking the abrupt steps typically associated with conventional ‘gate‐opening’ or ‘breathing’ effects. This smooth profile indicates that the structural reconfiguration of **Co‐Amor** is a gradual, kinetically controlled process rather than a sudden cooperative shift.

This dynamic adsorption behavior represents not merely real‐time uptake but a permanent reconstruction: the specific binding of CO_2_ supplies the enthalpic driving force that converts the disordered **Co‐Amor** matrix into crystalline **Co‐Mono1**. To confirm the chemoselectivity, we measured C_2_H_2_ and C_3_H_8_ isotherms at 273 K (Figure 4d and Figure S25). Notably, although **Co‐Mono1** takes up both C_2_H_2_ (53.5 cm^3^/g) and C_3_H_8_ (45.4 cm^3^/g), confirming that its channels are geometrically accessible to these gases, neither gas induces crystallization of **Co‐Amor** under prolonged exposure (Table S3). Adsorption capacity in the crystalline endpoint is therefore necessary but not sufficient to trigger the transition, which instead demands the specific C–H···O═C═O hydrogen‐bond complementarity unique to CO_2_. The adsorption event itself thus acts as the morphogenic trigger, guiding the material from its reactive hub to its crystalline sink.

Collectively, these adsorption dynamics reveal an apparent program of the cobalt formate structural landscape. By navigating the reversible 5‐phase‐transition cycle, we can effectively toggle the framework between various functional regimes. This direct correlation between the programmed structural phase and the resulting gas accessibility establishes a new pattern for minimalist MOFs, where complex, guest‐induced responsiveness is achieved through the strategic management of the amorphous‐crystalline interface. These isotherms demonstrate that the unique phase‐transition cycle is not simply a crystallographic curiosity but a mechanism for the on‐demand programming of gas accessibility and selectivity.

## 3 Discussion

In brief, we have demonstrated that the minimalist cobalt formate system can serve as a versatile platform for programmable structural interconversion among five topologically and compositionally distinct phases: a chiral hexagonal framework (**Co‐Hex**), a perovskite‐like trigonal framework (**Co‐Trig**), a guest‐exchanged diamondoid framework (**Co‐Mono1**), a monoclinic dihydrate (**Co‐Mono2**), and an amorphous phase (**Co‐Amor**). These phases are interconnected through a reversible 5‐phase‐transition cycle governed by thermal, humidity, guest, and adsorption stimuli. Central to this landscape is the amorphous **Co‐Amor** phase obtained by thermolysis, which functions not as a terminal degradation product but as a programmable reactive hub, a high‐energy, kinetically trapped reservoir from which multiple crystalline endpoints are selectively accessible. Cation‐specific hydrogen‐bonding templates dictate the reconstruction pathways: NH_4_^+^, acting as a tetrahedral hydrogen‐bond anchor, restores the original chiral hexagonal topology through a building‐block memory encoded in its four N–H···O contacts; Me_2_NH_2_^+^ directs the formation of the isostructural perovskite‐like **Co‐Trig** via an analogous mechanism. At the same time, the bulkier Et_3_NH^+^ redirects reconstruction toward the diamondoid framework, demonstrating explicit cation‐size control over product topology. Furthermore, CO_2_ adsorption selectively triggers recrystallization of **Co‐Amor** into **Co‐Mono1**, whereas other gas molecules, such as N_2_, H_2_, C_2_H_2_, and C_3_H_8_, do not. DFT calculations and in situ Raman spectroscopy reveal that this selectivity is rooted not in quadrupole strength or molecular size, but in a specific C–H···O═C═O hydrogen‐bond complementarity between the formate channel walls and the CO_2_ oxygen atoms, a molecular recognition mechanism that is uniquely satisfied by CO_2_ among the tested gas library. The incorporation of **Co‐Trig** into the phase‐transition network not only demonstrates the generality and modularity of the selective guest‐induced reconstruction principle, but also highlights the amorphous phase as a versatile structural hub. We believe this finding could be further expanded by systematically varying the templating cation, given that many other reported cations in cobalt formate frameworks are not included in this work. These findings establish a new paradigm for minimalist MOFs. By exploiting amorphous intermediates as divergent branching points that break kinetic barriers, even the simplest carboxylate frameworks can access a degree of multi‐pathway reconfigurability, encompassing amorphous‐to‐crystal, crystal‐to‐crystal, and guest‐directed topological switching, which rivals the structural complexity traditionally reserved for purpose‐designed flexible MOFs with far more elaborate scaffolds. Benefiting from reversible multiphase transition behavior and selective, guest‐responsive reconstruction, this system offers potential for developing colorimetric sensors for simple, visual detection of CO_2_, temperature, and other environmental parameters.

## 4 Materials and Methods

### 4.1 Chemicals and Synthesis

All chemicals and solvents in this work were commercially available (Sigma‐Aldrich) and used without further purification.

#### 4.1.1 Synthesis of **Co‐Hex** ([NH_4_][Co(HCOO)_3_])

Following a modified literature procedure, a methanol solution (8 mL) containing ammonium formate (NH_4_HCOO, 0.8 M) and formic acid (HCOOH, 0.4 M) was placed in a 20 mL test tube [22]. A separate methanol solution (8 mL) of cobalt perchlorate hexahydrate (Co(ClO_4_)_2_·6H_2_O, 0.1 M) was layered on top. Violet‐red bipyramidal crystals formed after 48 h at 25°C (yield: 48% based on Co). The crystals were washed with methanol (3 × 5 mL), soaked in acetone for 24 h, and dried under vacuum at 80°C for 12 h. Anal. Calcd for C_3_H_7_CoNO_6_ (MW = 212.02): C, 17.0; H, 3.33; N, 6.61. Found: C, 16.8; H, 3.38; N, 6.17.

#### 4.1.2 Synthesis of **Co‐Mono1** (Co(HCOO)_2_)

Following an adapted solvothermal method, cobalt nitrate hexahydrate (Co(NO_3_)_2_·6H_2_O, 0.25 M) was dissolved in N,N‐dimethylformamide (DMF, 8 mL) in a 30 mL screw‐cap glass vial. Formic acid (HCOOH, 7.8 mL) was added and the vial sealed. The mixture was heated at 40°C for 8 h, yielding pink hexagonal plates (yield: 58% based on Co) [29, 30]. The product was collected by filtration, washed with DMF (3 × 5 mL), soaked in acetone for 24 h (replacing the solvent every 8 h), and dried under vacuum at 180°C for 12 h. Anal. Calcd for C_2_H_2_CoO_4_ (MW = 148.97): C, 16.1; H, 1.35. Found: C, 16.6; H, 2.26. The elevated hydrogen content is attributed to residual adsorbed solvent. Thermogravimetric analysis (TGA, Figure S1b) confirms a mass loss of 1.6% before decomposition.

#### 4.1.3 Synthesis of **Co‐Mono2** (Co(HCOO)_2_·2H_2_O)

**Co‐Hex** or **Co‐Mono1** was spread in a thin layer on a glass Petri dish and exposed to humid air (25°C, relative humidity ∼60%, 48 h). The color changed from violet‐red (**Co‐Hex**) or pink (**Co‐Mono1**) to pale pink, indicating hydration. (yield: 98% based on Co). The resulting powder was collected and dried under vacuum at 50°C for 12 h. Anal. Calcd for C_2_H_6_CoO_6_ (MW = 184.99): C, 13.0; H, 3.27. Found: C, 13.0; H, 3.31.

#### 4.1.4 Synthesis of **Co‐Amor** (Co(HCOO)_2_)

**Co‐Hex** or **Co‐Mono2** (100 mg) was placed in a Schlenk tube and heated under vacuum at 200°C for 12 h. The sample color changed from violet‐red or pale pink to purple (Figure S3). The product was stored under N_2_ atmosphere to prevent moisture uptake. (yield: 89% based on Co). Anal. Calcd for C_2_H_2_CoO_4_ (MW = 148.97): C, 16.1; H, 1.35. Found: C, 15.4; H, 1.97. The elevated hydrogen content is attributed to rapid moisture uptake during the elemental analysis experiment. TGA (Figure S1d) confirms a mass loss of 4.3% before decomposition.

#### 4.1.5 Synthesis of **Co‐Trig** ([(CH_3_)_2_NH_2_][Co(HCOO)_3_])

Following a modified literature procedure, a mixture of CoCl_2_·6H_2_O (3.75 mmol), DMA (1.42 mL, 40% in water), formic acid (0.43 mL), DMF (30 mL), and H_2_O (30 mL) was transferred into a Teflon‐lined autoclave and heated at 140°C for 2 days [20, 31]. Bulk crystals formed after filtration of the solution and evaporation over 2 days (yield: 75% based on Co). The crystals were washed with methanol (3 × 5 mL), soaked in acetone for 24 h, and dried under vacuum at 80°C for 12 h. Anal. Calcd for C_5_H_11_CoNO_6_ (MW = 240.08): C, 25.0; H, 4.62; N, 5.83. Found: C, 25.1; H, 4.68; N, 5.77.

### 4.2 Phase Transition Methods

All phase transitions were monitored using time‐resolved powder X‐ray diffraction (PXRD) to confirm the completion of the transformation. Each pathway was repeated at least 3 times. Representative PXRD patterns for each pathway are provided in Figures S8–S19.

Pathway 1: from **Co‐Amor** to **Co‐Hex** (NH_4_^+^‐templated reconstruction). **Co‐Amor** (50 mg) was immersed in NH_4_HCOO/methanol (0.8 M, 10 mL) at 25°C. The mixture was left undisturbed for 24 h. The resulting violet‐red powder was collected by filtration, washed with methanol (3 × 5 mL), and dried under vacuum at 80°C for 12 h (see Figure S8).

Pathway 2a: from **Co‐Amor** to **Co‐Mono1** (CO_2_‐induced reconstruction). **Co‐Amor** (50 mg) was placed in a vial within a sealed chamber. The chamber was purged with high‐purity CO_2_ (99.9%, 1 atm) at 25°C and left for 48 h (see Figure S9).

Pathway 2b: from **Co‐Amor** to **Co‐Mono1** (Et_3_NH^+^‐induced reconstruction). **Co‐Amor** (50 mg) was immersed in (Et_3_NH)(HCOO)/methanol (0.8 M, 10 mL) at 25°C for 24 h. The product was collected by filtration, washed with methanol (3 × 5 mL), and dried under vacuum at 80°C for 12 h. (see Figure S10).

Pathway 3: from **Co‐Amor** to **Co‐Mono2** (humidity‐induced hydration). **Co‐Amor** (50 mg) was placed in a vial within a sealed chamber. The chamber was purged with ambient humid air (25°C, relative humidity ∼60%) for 8 h. The resulting pale pink solid was characterized by PXRD (see Figure S11).

Pathway 4: from **Co‐Hex** to **Co‐Amor** (thermal de‐templating). **Co‐Hex** (50 mg) was placed in a vial within a sealed chamber. The chamber was purged with N_2_ and was heated at 200°C under vacuum for 2 h (see Figure S12).

Pathway 5: from **Co‐Mono2** to **Co‐Amor** (thermal dehydration). **Co‐Mono2** (50 mg) was placed in a vial within a sealed chamber. The chamber was purged with N_2_ and was heated at 200°C under vacuum for 2 h (see Figure S13).

Pathway 6: from **Co‐Mono2** to **Co‐Hex** (NH_4_^+^‐templated reconstruction). Same procedure as Pathway 1, using **Co‐Mono2** as the starting material (see Figure S14).

Pathway 7: from **Co‐Hex** to **Co‐Mono2** (humidity‐induced hydration). Same procedure as Pathway 3, using **Co‐Hex** as the starting material and extending the exposure time to 24 h (see Figure S15).

Pathway 8: from **Co‐Mono1** to **Co‐Hex** (NH_4_^+^‐templated reconstruction). Same procedure as Pathway 1, using **Co‐Mono1** as the starting material (see Figure S16).

Pathway 9: from **Co‐Mono1** to **Co‐Mono2** (humidity‐induced hydration). Same procedure as Pathway 7, using **Co‐Mono1** as the starting material (see Figure S17).

Pathway 10: from **Co‐Mono2** to **Co‐Mono1** (Et_3_NH^+^‐induced reconstruction). Same procedure as Pathway 2b, using **Co‐Mono2** as the starting material (see Figure S18).

Pathway 11: from **Co‐Amor** to **Co‐Trig** (Me_2_NH_2_^+^‐templated reconstruction). **Co‐Amor** (50 mg) was immersed in Me_2_NH_2_HCOO/methanol (0.8 M, 10 mL) at 25°C. The mixture was left undisturbed for 24 h. The resulting red‐pink powder was collected by filtration, washed with methanol (3 × 5 mL), and dried under vacuum at 80°C for 12 h (see Figure S19a).

Pathway 12: from **Co‐Trig** to **Co‐Amor** (thermal de‐templating). Same procedure as Pathway 4, using **Co‐Trig** as the starting material (see Figure S19a).

Pathway 13: from **Co‐Mono1** to **Co‐Trig** (Me_2_NH_2_^+^‐templated reconstruction). Same procedure as Pathway 11, using **Co‐Mono1** as the starting material (see Figure S19b).

Pathway 14: from **Co‐Mono2** to **Co‐Trig** (Me_2_NH_2_^+^‐templated reconstruction). Same procedure as Pathway 11, using **Co‐Mono2** as the starting material (see Figure S19c).

Pathway 15: from **Co‐Trig** to **Co‐Mono2** (humidity‐induced hydration). Same procedure as Pathway 7, using **Co‐Trig** as the starting material (see Figure S19c).

Pathway 16: from **Co‐Hex** to **Co‐Trig** (Me_2_NH_2_^+^‐templated reconstruction). Same procedure as Pathway 11, using **Co‐Hex** as the starting material (see Figure S19d).

Pathway 17: from **Co‐Trig** to **Co‐Hex** (NH_4_^+^‐templated reconstruction). Same procedure as Pathway 1, using **Co‐Trig** as the starting material (see Figure S19d).

### 4.3 Characterization Techniques

Laboratory PXRD was performed using a Rigaku SmartLab 9 kW X‐ray Diffractometer operated at 40 kV and 40 mA with Cu K_α1_ radiation (λ = 1.54059 Å). C H N elemental analysis was acquired on an Elementar Vario Micro Select Elemental Analyzer. TGA experiments were performed on a Mettler Toledo TGA/DSC3+ unit under N_2_ flow of 50 mL/min. A known amount of sample was added to an alumina pan and heated from 30°C to 400°C at a heating rate of 5°C/min. The corresponding TGA curve and its first derivative were analyzed simultaneously. FT‐IR spectra were recorded in the range of 4000–400 cm^−1^ on a Thermo Scientific Nicolet iS50 Fourier Transform Infrared (FTIR) Spectrometer at 25°C. TPD‐MS analysis was performed on a Micromeritics AutoChem III 2930 chemisorption analyzer. Approximately 30 mg of sample was placed in a quartz U‐tube and purged with ultrahigh‐purity helium (99.999%) at a flow rate of 50 mL/min at 30°C for 30 min to remove surface‐adsorbed impurities. The temperature was then ramped to 300°C at a heating rate of 3°C/min under continuous helium flow. At the same time, the desorption profiles were recorded simultaneously by a thermal conductivity detector (TCD) and an online mass spectrometer (MS). Scanning electron micrographs were acquired using a TESCAN CLARA microscope operating at an accelerating voltage of 2 kV and a working distance of 4–6 mm. Before measurement, the samples were uniformly dispersed onto conductive carbon tape and sputter‐coated with gold for 1–2 min to enhance surface conductivity.

N_2_ (at 77 K), H_2_ (at 77 K), CO_2_ (at 273 K), C_2_H_2_ (at 273 K), and C_3_H_8_ (at 273 K) gas sorption isotherms up to 1 bar were measured and analyzed using a Micromeritics ASAP 2020 Plus Micropore Analyzer. All sorption isotherms were obtained using high‐purity gases (N_2_: 99.999%; H_2_: 99.999%, CO_2_: 99.9%; C_2_H_2_: 99.9%; C_3_H_8_: 99.9%). Before the sorption analysis, a sample was loaded into a sample tube and subjected to a vacuum of 10^−3^ Torr at 80°C (**Co‐Hex**), 80°C (**Co‐Trig**), 160°C (**Co‐Mono1**), 80°C (**Co‐Mono2**), and 160°C (**Co‐Amor**) for 12 h, respectively. Pore width measurements were calculated based on the Horvath–Kawazoe (Original) method.

### 4.4 Synchrotron PXRD

Synchrotron PXRD measurements were collected on the 19A beamline at the National Synchrotron Radiation Research Center (Hsinchu, Taiwan, China). The incident X‐ray energy was set at 20 keV with a wavelength of 0.61992 Å. Zero error: 0.0006. The synchrotron PXRD data were analyzed via Pawley profile fit using TOPAS V7.0 software.

### 4.5 X‐ray Pair Distribution Function (XPDF)

XPDF measurements were collected on beamline ID31 of the European Synchrotron Radiation Facility (ESRF). The incident X‐ray energy was set at 75.051 keV with a wavelength of 0.16520 Å. Simulated X‐ray pair distribution functions (XPDF) of **Co‐Hex**, **Co‐Mono1**, and **Co‐Mono2** were calculated from the reported crystal structures using CrystalMaker V11.0 software with a rebinning step of 0.02 Å.

### 4.6 Raman Spectroscopy Measurements

In situ and ex situ Raman spectroscopy measurements were conducted utilizing a modified gas flow chamber adapted from a Gaoss Union cell. The metal–organic framework samples were deposited directly onto a glassy carbon substrate positioned inside the chamber without the addition of any electrolyte. Spectra were acquired using a Renishaw Micro Raman Spectroscopy System equipped with a diode laser source (λ = 785 nm) operating at 1% power. The optical probe was precisely focused onto the solid sample surface through a transparent quartz window.

For the in situ CO_2_ adsorption experiments on **Co‐Mono1**, high‐purity CO_2_ gas was continuously introduced into the chamber at a constant flow rate of 20 mL/min, regulated by a mass flow controller. Conversely, owing to the prolonged induction time required for the structural reconstruction and instrument time constraints, ex situ measurements were performed for **Co‐Amor**. In these ex situ Raman measurements, the same spatial location on the sample was irradiated before and after the CO_2_ exposure to ensure spectral consistency and accurately track local structural evolution.

### 4.7 Density Functional Theory Calculations

Density functional theory (DFT) calculations were performed utilizing the projector augmented‐wave method as implemented in the Vienna Ab initio Simulation Package (VASP). The exchange‐correlation functional of Perdew, Burke, and Ernzerhof within the generalized gradient approximation was employed. Dispersion interactions were incorporated via Grimme's D3 empirical correction scheme. A kinetic energy cutoff of 520 eV for the plane wave basis set was applied, and the criterion for electronic self‐consistency was set to 1.0 × 10^−5^ eV. Structural optimizations proceeded until the residual forces on all unconstrained atoms fell below 0.05 eV/Å. To accurately describe the strongly correlated d electrons of the cobalt centers, the Hubbard U correction was applied with an effective U value of 3.3 eV. To determine the most stable adsorption configuration, CO_2_ molecules were introduced at multiple candidate sites inside the pores of **Co‐Mono1** before full structural relaxation, and the geometry yielding the minimum total energy was selected.

## Author Contributions

Conceptualization: T.W.B.L., C.L., W.‐Y.Y., Z.T. Methodology: Z.T., J.Li, J.Lin. Investigation: Z.T., J.Li, J.Lin, T.C. Writing – original draft: T.W.B.L., C.L., W.‐Y.Y., Z.T. Writing – review & editing: T.W.B.L., C.L., W.‐Y.Y., Z.T.

## Funding

National Natural Science Foundation of China (22522207 and 22403080). Hong Kong Research Grants Council (15305722, 15301521, C5081‐21E, and C5057‐24E). The University Grants Committee of The Hong Kong Polytechnic University (PolyU) (P0058154 and P0047841).

## Conflicts of Interest

The authors declare no conflicts of interest.

## Supporting information




**Supporting File**: advs77629‐sup‐0001‐SuppMat.docx.

## Data Availability

The data that supports the findings of this study are available in the supplementary material of this article.
