# Supplementary material for: Programmable Guest‐Induced 5‐Phase‐Transition Topological Reconstructions of Cobalt Formate Metal–Organic Frameworks
Source: Adv Sci (Weinh). 2026 Sep 11:e77629. Online ahead of print. doi: 10.1002/advs.77629 (PMC13564190; doi:10.1002/advs.77629)
Supplement: Supplementary file 1 [file ADVS-9999-e77629-s001.docx]

**Supplementary Materials for**

**Programmable Guest-Induced 5-Phase-Transition Topological Reconstructions of Cobalt Formate Metal–Organic Frameworks**

Zhiyu Tao et al.

Corresponding author: wing-yiu.yu@polyu.edu.hk, cong.lin@polyu.edu.hk, benedict.tw.lo@polyu.edu.hk

**The PDF file includes:**

Figures S1 to S25

Tables S1 to S3

**Figure S1.** Thermogravimetric analysis (TGA) curves of the five cobalt formate MOFs: (**a**) **Co-Hex**; (**b**) **Co-Mono1**; (**c**) **Co-Mono2**; (**d**) **Co-Amor**; (**e**) **Co-Trig**. **Co-Hex**: A distinct weight loss of 28.3% is observed between 175 and 255 °C. This value agrees with the theoretical mass loss for the release of one equivalent of ammonium formate template (NH_4_HCOO, calculated ~29.7%). **Co-Mono1**: The sample exhibits negligible mass loss (~1.6%) up to 300 °C, indicating that the drying process successfully removed almost all guest molecules. **Co-Mono2**: Two weight loss steps are observed at 75 °C (13.0%) and 160 °C (11.1%), totaling 24.1%. This value is slightly higher than the theoretical weight loss for two coordinated H_2_O (calculated ~19.5%). We attribute this excess weight loss to the rapid adsorption of surface moisture from the air. **Co-Amor**: A gradual, minor mass loss (4.3%) is observed before decomposition, which is similarly attributed to the rapid adsorption of atmospheric moisture. **Co-Trig**: A weight loss of 36.2% is observed between 130 and 225 °C, which agrees with the theoretical mass loss for the release of one equivalent of dimethylamine formate template per formula unit (Me_2_NH_2_HCOO, calculated ~37.9%). All five phases undergo final framework decomposition at approximately 300 °C, yielding cobalt species (oxides/metallic Co) and carbonaceous residues.

**Figure S2.** Scanning electron micrographs of the synthesized cobalt formate MOFs: (**a**) bipyramidal cubic for **Co-Hex**; (**b**) hexagonal plates for **Co-Mono1**; (**c**) truncated cubic for **Co-Mono2**; (**d**) spherical particles for **Co-Amor**; (**e**) cuboidal particles for **Co-Trig**.

**Figure S3**. Optical photographs of the five cobalt formate MOFs.

**Figure S4.** FTIR spectra of **Co-Hex**, **Co-Mono1**, **Co-Mono2**, **Co-Amor**, and **Co-Trig**. Assignments: ν(OCO)_as_ at 1591 (cm^-1^); ν(OCO)_s_ 1373 (cm^-1^); Co–O lattice vibrations at 570/807 (cm^-1^).

**Figure S5.** Simulated XPDF of **Co-Hex**.

**Figure S6.** Simulated XPDF of **Co-Mono1**.

**Figure S7.** Simulated XPDF of **Co-Mono2**.

**Figure S8.** PXRD tracking Pathway 1: Phase transition from **Co-Amor** to **Co-Hex**.

**Figure S9.** PXRD tracking Pathway 2a: Phase transition from **Co-Amor** to **Co-Mono1**.

**Figure S10.** PXRD tracking Pathway 2b: Phase transition from **Co-Amor** to **Co-Mono1**.

**Figure S11.** PXRD tracking Pathway 3: Phase transition from **Co-Amor** to **Co-Mono2**.

**Figure S12.** PXRD tracking Pathway 4: Phase transition from **Co-Hex** to **Co-Amor**.

**Figure S13.** PXRD tracking Pathway 5: Phase transition from **Co-Mono2** to **Co-Amor**.

**Figure S14.** PXRD tracking Pathway 6: Phase transition from **Co-Mono2** to **Co-Hex**.

**Figure S15.** PXRD tracking Pathway 7: Phase transition from **Co-Hex** to **Co-Mono2**.

**Figure S16.** PXRD tracking Pathway 8: Phase transition from **Co-Mono1** to **Co-Hex**.

**Figure S17.** PXRD tracking Pathway 9: Phase transition from **Co-Mono1** to **Co-Mono2**.

**Figure S18.** PXRD tracking Pathway 10: Phase transition from **Co-Mono2** to **Co-Mono1**.

**Figure S19.** Phase transition pathways involving **Co-Trig**: (**a**) Pathway 11 and 12: heat treatment of **Co-Trig** at 200 °C and Me_2_NH_2_^+^ immersion of **Co-Amor**. (**b**) Pathway 13: Me_2_NH_2_^+^ immersion of **Co-Mono1**. (**c**) Pathway 14 and 15: exposure to humid air of **Co-Trig** and Me_2_NH_2_^+^ immersion of **Co-Mono2**. (**d**) Pathway 16 and 17: NH_4_^+^ immersion of **Co-Trig** and Me_2_NH_2_^+^ immersion of **Co-Hex**.

**Figure S20**. Raman spectroscopy and DFT calculations: (**a**) in situ Raman spectroscopy of **Co-Mono1** and (b) ex situ Raman spectroscopy of **Co-Amor** upon exposure to CO_2_ (25 °C, 1 bar). (**c**) DFT calculated structure showing the adsorbed CO_2_ species within the channel of **Co-Mono1**.

**Figure S21**. PXRD patterns of **Co-Amor** after exposure to N_2_, H_2_, C_2_H_2_, and C_3_H_8_ (25 °C, 48 h, 1 atm). All patterns retain the broad, featureless profile of the pristine amorphous phase, confirming that none of these gases induces crystallization.

**Figure S22.** TPD-MS profiles of four cobalt formate MOFs: (**a**) **Co-Hex**; (**b**) **Co-Mono1**; (**c**) **Co-Mono2**; (**d**) **Co-Amor**.

**Figure S23.** Scanning electron micrographs of **Co-Hex** and **Co-Amor** tracking various phase transitions as labeled.

**Figure S24.** Gas adsorption isotherms**.** Adsorption (filled circles) and desorption (open circles) profiles for (**a**) N_2_ at 77 K and (**b**) CO_2_ at 273 K. Data series are color-coded as follows: orange for **Co-Mono1**, green for **Co-Trig,** and dark blue for **Co-Hex**.

**Figure S25**. Gas adsorption isotherms of C_3_H_8_ at 273 K. Filled circles represent adsorption, and open circles represent desorption. Data series are color-coded as follows: orange for **Co-Mono1**, cyan for **Co-Amor**, dark blue for **Co-Hex**, and yellow for **Co-Mono2**.

**Table S1.** Elemental analysis results of compounds **Co-Hex**, **Co-Mono1**, **Co-Mono2,** **Co-Amor** and **Co-Trig**.

| Compound | Starting compound | N(%) | C(%) | H(%) |
| --- | --- | --- | --- | --- |
| **Co-Hex** | [NH_4_][Co(HCOO)_3_] | 6.17 (6.61) | 16.8 (17.0) | 3.38 (3.33) |
| **Co-Mono1** | Co(HCOO)_2_ | 0 (0) | 16.6 (16.1) | 2.26 (1.35) |
| **Co-Mono2** | Co(HCOO)_2_·2H_2_O | 0.44 (0) | 13.0 (13.0) | 3.31 (3.27) |
| **Co-Amor** | Co(HCOO)_2_ | 0 (0) | 15.4 (16.1) | 1.97 (1.35) |
| **Co-Trig** | [(CH_3_)_2_NH_2_][Co(HCOO)_3_] | 5.77 (5.83) | 25.1 (25.0) | 4.68 (4.62) |

Note: Calculated data are in parentheses.

**Table S2.** Crystallographic parameters of **Co-Hex**, **Co-Mono1** and **Co-Mono2** from the Pawley refinement of synchrotron PXRD data.

|  | **Co-Hex** | **Co-Mono1** | **Co-Mono2** |
| --- | --- | --- | --- |
| X-ray energy (keV) | 20 | 20 | 20 |
| Beamline | 19A | 19A | 19A |
| Wavelength (Å) (synchrotron) | 0.61992 | 0.61992 | 0.61992 |
| 2θ - zero point (°) | 0.0006 | 0.0006 | 0.0006 |
| Space group | *P*6_3_22 | *P*2_1_/c | *P*2_1_/c |
| Crystal system | Hexagonal | Monoclinic | Monoclinic |
| *a* (Å) | 7.306 | 11.324 | 8.682 |
| *b* (Å) | 7.306 | 9.950 | 7.171 |
| *c* (Å) | 8.186 | 18.347 | 9.278 |
| *β* (°) | 90° | 127.193° | 97.247° |
| *V* (Å^3^) | 378.481 | 1655.791 | 572.804 |
| 2θ range for refinement (°) | 5–60 | 5–60 | 5–60 |
| Number of parameters | 29 | 31 | 31 |
| Number of *hkls* | 387 | 7439 | 5130 |
| Refinement methods | Pawley | Pawley | Pawley |
| *R_wp_*/*R_exp_*/*R_p_* (%) | 7.639/2.207/5.135 | 12.406/2.272/5.931 | 6.431/3.351/3.695 |
| *Gof* | 3.460 | 5.459 | 1.918 |

Note: *R_wp_*: weighted profile; *R_exp_*: expected; *R_p_*: profile; *Gof*: goodness-of-fit.

**Table S3.** Structural outcome of **Co-Amor** upon exposure to different probe gases.

| **Gas** | **Quadrupole moment (×10^-40^ C·m^2^)** | **Kinetic diameter (Å)** | **H-bond role** | **Phase change?** |
| --- | --- | --- | --- | --- |
| CO_2_ | 4.30 | 3.3 | Acceptor | **Co-Amor** to **Co-Mono1** |
| C_2_H_2_ | 7.50 | 3.3 | Donor | No |
| N_2_ | 1.50 | 3.6 | Neither | No |
| H_2_ | 0.66 | 2.9 | Neither | No |
| C_3_H_8_ | - | 4.3 | Neither | No |

Note: Gas exposure experiments were determined by PXRD (25 °C, 48 h, 1 atm). For each gas, the quadrupole moment (Q), kinetic diameter, H-bond role, and phase transition results are listed for comparison.
